# Evolução de 16 Anos da Doença de Chagas Aguda Transmitida por Via Oral: Relato de Caso Autóctone com Progressão para Cardiomiopatia Chagásica

**DOI:** 10.36660/abc.20250817

**Published:** 2026-06-10

**Authors:** Monica Regina Hosannah Silva e Silva, Jorge Augusto de Oliveira Guerra, Jessica Vanina Ortiz, Matheus Martins Monteiro, Katia do Nascimento Couceiro, Maria das Graças Vale Barbosa Guerra, João Marcos Bemfica Barbosa Ferreira

**Affiliations:** 1 Universidade do Estado do Amazonas Escola de Ciências da Saúde Manaus AM Brasil Universidade do Estado do Amazonas – Programa de Pós-Graduação em Medicina Tropical, Escola de Ciências da Saúde, Manaus, AM – Brasil; 2 Fundação de Hematologia e Hemoterapia do Amazonas Manaus AM Brasil Fundação de Hematologia e Hemoterapia do Amazonas, Manaus, AM – Brasil; 3 Fundação de Medicina Tropical Doutor Heitor Vieira Dourado Manaus AM Brasil Fundação de Medicina Tropical Doutor Heitor Vieira Dourado, Manaus, AM – Brasil; 4 Universidade Federal do Amazonas Manaus AM Brasil Universidade Federal do Amazonas, Manaus, AM – Brasil; 5 Beneficência Portuguesa de São Paulo São Paulo SP Brasil Beneficência Portuguesa de São Paulo, São Paulo, SP – Brasil

**Keywords:** Doença de Chagas, Cardiomiopatia Chagásica, Insuficiência Cardíaca

## Introdução

A doença de Chagas (DC) é causada pelo Trypanosoma cruzi, um protozoário com ciclo de vida complexo encontrado em dezenas de espécies de insetos triatomíneos (barbeiros) e mamíferos.^1^ A Organização Mundial da Saúde (OMS) incluiu a DC entre as doenças tropicais negligenciadas em 2006.^2^

Os seres humanos podem se infectar por meio do contato com fezes de insetos contendo T. cruzi ou por outros mecanismos, incluindo a ingestão de alimentos contaminados. A apresentação clínica da DC varia entre suas duas fases: aguda e crônica. Na fase aguda, os indivíduos geralmente são oligossintomáticos, apresentando sinais e sintomas inespecíficos. No entanto, uma minoria pode desenvolver manifestações mais graves, como hepatoesplenomegalia, miocardite ou meningoencefalite. A fase crônica compreende três formas clínicas bem definidas: indeterminada, cardíaca e digestiva.^1–6^

Por muitos anos, a região Amazônica foi considerada livre de DC endêmica, mas atualmente é a área com o maior número de casos agudos, em grande parte devido a surtos de transmissão oral associados ao consumo de sucos de frutos de palmeira, especialmente o açaí (*Euterpe oleraceae*). No estado do Amazonas, alguns casos de cardiopatia chagásica crônica já foram diagnosticados.^1,3^ Este relato de caso documenta um seguimento longitudinal de 16 anos do primeiro caso autóctone de DC aguda acompanhado pelo Grupo de Estudo da Doença de Chagas da Fundação de Medicina Tropical Dr. Heitor Vieira Dourado (FMT-HVD), no Amazonas. A evolução clínica do paciente foi acompanhada desde o diagnóstico inicial na fase aguda, passando por diversas manifestações fenotípicas da forma cardíaca, culminando em melhora clínica.

## Relato de caso

Um homem de 31 anos, natural de Manaus, Amazonas (Brasil), foi admitido na FMT-HVD, um centro terciário de referência, em 2007, com história de febre, palpitações, dispneia e dor torácica após ingerir suco de açaí.

A DC aguda foi diagnosticada por exame parasitológico direto utilizando gota espessa. O eletrocardiograma (ECG) mostrou extrassístoles ventriculares frequentes, e o ecocardiograma transtorácico (ETT) demonstrou disfunção sistólica leve do ventrículo esquerdo, com fração de ejeção de 50%. O paciente recebeu benznidazol como tratamento etiológico para DC e iniciou terapia padrão para insuficiência cardíaca (IC), incluindo um inibidor da enzima conversora de angiotensina, um betabloqueador e um diurético de alça. No entanto, após a alta hospitalar, ele interrompeu o benznidazol após 30 dias, não completando o regime obrigatório de 60 dias. Meses depois, seus exames cardíacos haviam normalizado, permitindo a retirada gradual das medicações para IC.

Posteriormente, o paciente manteve acompanhamento cardiológico irregular. Em 2010, retornou à FMT-HVD relatando dispneia aos médios esforços e palpitações. A sorologia (ELISA) para T. cruzi foi negativa, assim como dois exames de xenodiagnóstico. O ECG de repouso mostrou ritmo sinusal com extrassístoles ventriculares isoladas e repolarização normal. Tanto o ETT quanto a ressonância magnética cardíaca estavam normais, enquanto o Holter de 24 horas revelou três episódios de taquicardia ventricular não sustentada. Um esofagograma demonstrou esvaziamento esofágico completo. Naquele momento, foi diagnosticada a forma arritmogênica da cardiomiopatia chagásica crônica. O tratamento com amiodarona resultou em melhora clínica.

O paciente permaneceu assintomático até 2015, quando as palpitações retornaram. Nesse momento, amiodarona e captopril foram reintroduzidos. Reavaliações subsequentes, incluindo sorologia negativa para *T. cruzi* (ELISA) e exames cardiológicos (ECG e teste ergométrico), apresentaram resultados dentro da normalidade.

Em 2018, 2019 e 2022, o paciente retornou para consultas de seguimento, durante as quais permaneceu clinicamente estável, sem alterações significativas nos exames cardiológicos, conforme resumido na Tabela 1.

**Tabela 1:** Exames cardiológicos realizados no paciente durante o período de seguimento, de 2007 a 2023

Ano	Exame	Principais alterações
2007	Eletrocardiograma	Ritmo sinusal; contração ventricular prematura
	Ecocardiograma	Disfunção sistólica leve do ventrículo esquerdo; FE 50%
2010	Eletrocardiograma	Ritmo sinusal; contração ventricular prematura; repolarização normal
	Ecocardiograma	FE preservada, dimensões intracavitárias normais
	Ressonância magnética cardíaca	Aumento da cavidade do ventrículo esquerdo sem sinais de fibrose
	Holter 24 horas	Ritmo sinusal, 28 190 contrações ventriculares isoladas e pareadas, 3 episódios de taquicardia ventricular não sustentada
2018	Eletrocardiograma	Ritmo sinusal com contrações ventriculares frequentes
	Ecocardiogramas	Leve aumento das dimensões intracavitárias com DDVE 60 mm, FEVE (Simpson) 50%, com disfunção sistólica global leve
	Holter 24 horas	7668 contrações ventriculares isoladas, interpoladas, bigeminadas, trigeminadas e polimórficas
	Ressonância magnética cardíaca	Aumento da cavidade do ventrículo esquerdo sem sinais de fibrose
2019	Eletrocardiograma	Ritmo sinusal, alteração de repolarização em parede inferior
	Ecocardiograma	Aumento das dimensões intracavitárias; DDVE 60 mm; FEVE (Simpson) 53%
2022	Eletrocardiograma	Ritmo sinusal, sem alterações
	Ecocardiograma	Redução acentuada da FE (FEVE 37%) e disfunção sistólica e diastólica moderada do ventrículo esquerdo
	Holter 24 horas	Ritmo sinusal com 193 extrassístoles ventriculares isoladas, interpoladas, bigeminadas, trigeminadas e monomórficas
2023	Eletrocardiograma	Ritmo sinusal, repolarização normal
	Ecocardiograma	FEVE (Simpson) 52%; DDVE 60 mm
	Holter 24 horas	Ritmo sinusal, 444 contrações ventriculares isoladas

DDVE: diâmetro diastólico do ventrículo esquerdo; FEVE: fração de ejeção do ventrículo esquerdo.

**Figura 1:**
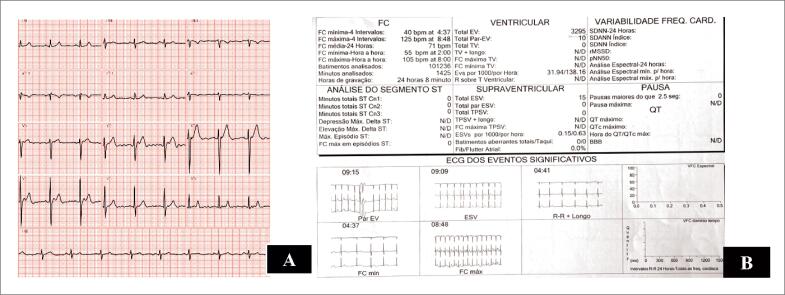
A) Eletrocardiograma realizado em 2020; B) Holter de 24 horas realizado em 2020.

Durante esse período, os testes sorológicos para *T. cruzi* (ELISA: resultado 1,3; cut-off 0,27; IFI: 1/160) foram reagentes (Figura 1).

Na reavaliação de 2022, o paciente apresentou sinais clínicos de IC, incluindo dispneia aos esforços e ortopneia. Houve uma redução significativa da fração de ejeção do ventrículo esquerdo (FEVE 37%), indicando disfunção sistólica moderada. Os testes sorológicos (ELISA e IFI) permaneceram reagentes. O Holter de 24 horas mostrou ritmo sinusal com 193 extrassístoles ventriculares isoladas, interpoladas, em bigeminismo, trigeminismo e monomórficas. Foi iniciada terapia medicamentosa guiada por diretrizes (GDMT), incluindo carvedilol 25 mg duas vezes ao dia, enalapril 10 mg/dia, espironolactona 25 mg/dia e dapagliflozina 10 mg/dia. Apesar de um mês de tratamento, ele continuou apresentando taquicardia e dispneia paroxística noturna, levando à adição de furosemida 40 mg/dia e digoxina 0,25 mg/dia.

**Figura 2:**
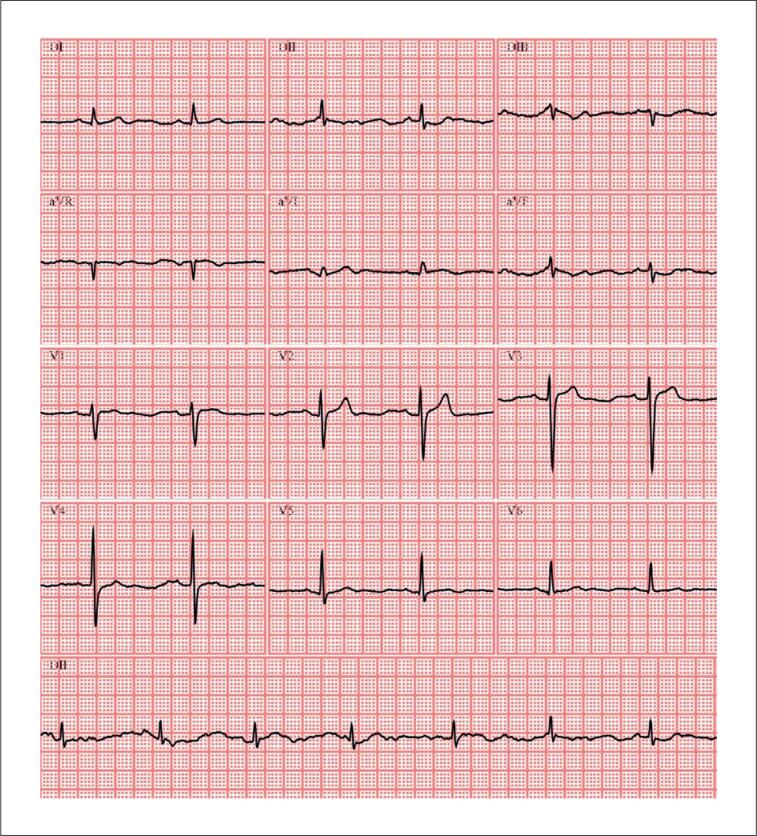
Eletrocardiograma pós-tratamento.

Em 2023, durante a continuidade do tratamento para IC, novos exames foram realizados. O ECG mostrou ritmo sinusal com frequência cardíaca de 69 bpm. O ETT demonstrou FEVE de 52% (Simpson) e diâmetro diastólico do ventrículo esquerdo (DDVE) de 60 mm, preenchendo os critérios para IC com fração de ejeção melhorada (Figuras 2 e 3).

**Figura 3:**
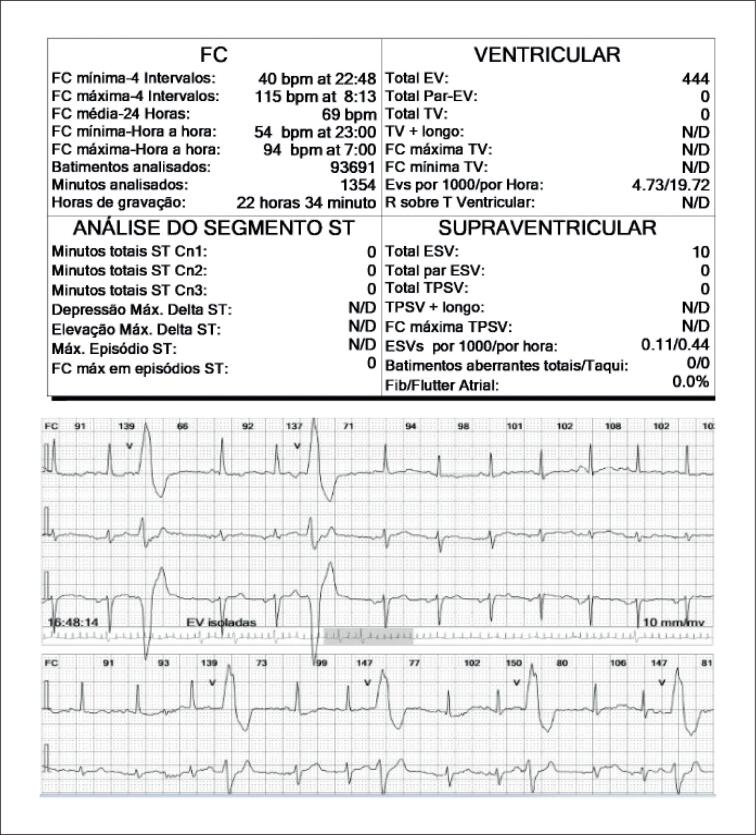
Exame de Holter de 24 horas realizado em 2023.

Durante o período de seguimento, o paciente também foi diagnosticado com leishmaniose cutâneo-mucosa e tuberculose pulmonar, ambas tratadas conforme as diretrizes clínicas. O histórico dos exames cardiológicos realizados ao longo desse acompanhamento prolongado está resumido na Tabela 1.

## Discussão

Este caso representa a primeira progressão documentada para CMDC no estado do Amazonas, Brasil. Ao longo de 16 anos de acompanhamento, o paciente inicialmente desenvolveu uma forma arritmogênica da CMDC, progredindo posteriormente para IC com fração de ejeção reduzida e, mais tarde, para IC com fração de ejeção recuperada. Estudos prévios demonstraram que o tratamento antiparasitário reduz tanto a proporção quanto o risco de IC sintomática.^7–9^

No entanto, a eficácia da terapia antiparasitária na eliminação completa do parasita é variável, e uma maior carga parasitária está associada a respostas sorológicas mais intensas. Apesar de mais de 10 anos de sorologia negativa neste caso, as manifestações cardíacas persistiram, sugerindo atividade contínua da doença. O longo período de sorologia negativa seguido por resultados reativos posteriores é um achado relevante. A variabilidade diagnóstica na região Amazônica é bem reconhecida, com alta frequência de resultados falso-negativos.^10^

Além disso, não se pode descartar a possibilidade de reinfecção, já que o paciente permaneceu em área endêmica. Importante destacar que ele não completou o tratamento tripanocida com benznidazol. Em um estudo prévio realizado na Amazônia brasileira, envolvendo 179 pacientes que completaram o tratamento com benznidazol, aproximadamente 30% permaneceram soropositivos após sete anos; entretanto, apenas 5,2% apresentaram alterações eletrocardiográficas ou ecocardiográficas sugestivas de CMDC.^11^

Na fase inicial da cardiomiopatia chagásica, as contrações ventriculares prematuras são a arritmia predominante. À medida que a doença progride e surge IC, cerca de 99% dos pacientes apresentam contrações ventriculares frequentes, multiformes, pareadas e episódios de taquicardia ventricular não sustentada. De forma consistente com esse padrão, o paciente desenvolveu palpitações e arritmias ventriculares, sendo classificado como portador de cardiomiopatia chagásica crônica com fenótipo arritmogênico.^3,8,9^

Quinze anos após o diagnóstico, o paciente apresentou IC caracterizada por fração de ejeção reduzida (FEVE 37%), compatível com a progressão natural da IC inadequadamente tratada, a qual está associada a maior risco cardiovascular e pior qualidade de vida. Sua evolução clínica está alinhada aos achados de uma revisão sistemática e meta-análise que demonstraram uma probabilidade cumulativa de cardiomiopatia de aproximadamente 17% em 10 anos e 31% em 20 anos entre indivíduos com DC.^12^

A adesão à terapia medicamentosa guiada por diretrizes – incluindo inibidores da enzima conversora de angiotensina, betabloqueadores, antagonistas do receptor mineralocorticoide e inibidores de SGLT2 – resultou em melhora da função ventricular esquerda e redução das arritmias ventriculares.^13^

A ausência de fibrose à ressonância magnética cardíaca, apesar da disfunção ventricular, é um achado relevante. Estudos prévios da Amazônia brasileira mostraram que 58,3% dos pacientes com DC aguda apresentam fibrose miocárdica à ressonância. Entretanto, esses pacientes geralmente apresentavam bom prognóstico e ausência de sintomas de IC.^14^ Esse achado sugere que, no paciente descrito, outro mecanismo fisiopatológico — como disfunção autonômica — pode ter contribuído para a disfunção ventricular, o que poderia explicar a rápida melhora com o tratamento farmacológico.

Em uma revisão sistemática que avaliou o risco de cardiomiopatia crônica entre pacientes com formas aguda ou indeterminada da DC, séries de casos de diversas regiões da América Latina indicaram uma incidência anual estimada de cardiomiopatia de 4,6% entre indivíduos com infecção aguda, sugerindo uma taxa relativamente baixa de progressão para a forma crônica.^9^

Este caso evidencia como doenças negligenciadas, como a DC, afetam de forma desproporcional populações economicamente vulneráveis. Apesar da disponibilidade de recursos diagnósticos e terapêuticos para diversas condições, grande parte da população amazônica continua exposta a doenças como a DC, que se perpetuam em contextos de pobreza e ampliam desigualdades sociais.^3^

Em conclusão, há uma necessidade urgente de ampliar o conhecimento sobre a DC na Amazônia, bem como aprimorar o diagnóstico e o tratamento da IC, a fim de reduzir desigualdades no acesso à saúde e abordar causas tratáveis de IC na população amazônica.

## Data Availability

Os conteúdos subjacentes ao texto da pesquisa estão contidos no manuscrito.

## References

[1] Dias JC, Ramos AN, Gontijo ED, Luquetti A, Shikanai-Yasuda MA, Coura JR (2016). 2 nd Brazilian Consensus on Chagas Disease, 2015. Rev Soc Bras Med Trop.

[2] World Health Organization (2025). Chagas Disease (American Trypanosomiasis) [Internet].

[3] Marin-Neto JA, Rassi A, Oliveira GMM, Correia LCL, Ramos NA, Luquetti AO (2023). SBC Guideline on the Diagnosis and Treatment of Patients with Cardiomyopathy of Chagas Disease - 2023. Arq Bras Cardiol.

[4] Barbosa MGV, Ferreira JMBB, Arcanjo ARL, Santana RAG, Magalhães LKC, Magalhães LKC (2015). Chagas Disease in the State of Amazonas: History, Epidemiological Evolution, Risks of Endemicity and Future Perspectives. Rev Soc Bras Med Trop.

[5] Ortiz JV, Pereira BVM, Couceiro KN, Silva MRHS, Doria SS, Silva PRL (2019). Cardiac Evaluation in the Acute Phase of Chagas' Disease with Post-Treatment Evolution in Patients Attended in the State of Amazonas, Brazil. Arq Bras Cardiol.

[6] Santana RAG, Guerra MGVB, Sousa DR, Couceiro K, Ortiz JV, Oliveira M (2019). Oral Transmission of Trypanosoma Cruzi, Brazilian Amazon. Emerg Infect Dis.

[7] Albajar PV, Laredo SV, Terrazas MB, Coura JR (2003). Dilated Cardiomyopathy in Patients with Chronic Chagasic Infection: Report of Two Fatal Autochthonous Cases from Rio Negro, State of Amazonas, Brazil. Rev Soc Bras Med Trop.

[8] Barbosa-Ferreira JM, Guerra JAO, Santana FS, Magalhães BML, Coelho LIARC, Barbosa MGV (2010). Cardiac Involvement in Acute Chagas' Disease cases in the Amazon Region. Arq Bras Cardiol.

[9] Chadalawada S, Sillau S, Archuleta S, Mundo W, Bandali M, Parra-Henao G (2020). Risk of Chronic Cardiomyopathy among Patients with the Acute Phase or Indeterminate Form of Chagas Disease: A Systematic Review and Meta-Analysis. JAMA Netw Open.

[10] Sáez-Alquezar A, Junqueira ACV, Durans AM, Guimarãe AV, Corrêa JÁ, Borges-Pereira J (2021). Geographical Origin of Chronic Chagas Disease Patients in Brazil Impacts the Performance of Commercial Tests for Anti-T. Cruzi IgG. Mem Inst Oswaldo Cruz.

[11] Pinto AYN, Valente VC, Coura JR, Valente SAS, Junqueira ACV, Santos LC (2013). Clinical Follow-Up of Responses to Treatment with Benznidazol in Amazon: A Cohort Study of Acute Chagas Disease. PLoS One.

[12] Romero J, Velasco A, Pisani CF, Alviz I, Briceno D, Díaz JC (2021). Advanced Therapies for Ventricular Arrhythmias in Patients with Chagasic Cardiomyopathy: JACC State-of-the-Art Review. J Am Coll Cardiol.

[13] McDonagh TA, Metra M, Adamo M, Gardner RS, Baumbach A, Böhm M (2023). 2023 Focused Update of the 2021 ESC Guidelines for the Diagnosis and Treatment of Acute and Chronic Heart Failure. Eur Heart J.

[14] Couceiro KDN, Ortiz JV, Silva MRHS, Sousa DRT, Andrade RC, Brandão ARJ (2022). Myocardial Injury in Patients with Acute and Subacute Chagas Disease in the Brazilian Amazon Using Cardiovascular Magnetic Resonance. J Am Heart Assoc.

