# Supplementary material for: Evolução de 16 Anos da Doença de Chagas Aguda Transmitida por Via Oral: Relato de Caso Autóctone com Progressão para Cardiomiopatia Chagásica
Source: Arq Bras Cardiol. 2026 Jun 10;123(5):e20250817. [Article in Portuguese] doi: 10.36660/abc.20250817 (PMC13399176; doi:10.36660/abc.20250817)
Supplement: Translation

# Translation: 16-Year Evolution of Acute Orally Transmitted Chagas Disease: Report of an Autochthonous Case Progressing to Chagasic Cardiomyopathy

**DOI:** 10.36660/abc.20250817i

**Authors:** Monica Regina Hosannah Silva e Silva, Jorge Augusto de Oliveira Guerra, Jessica Vanina Ortiz, Matheus Martins Monteiro, Katia do Nascimento Couceiro, Maria das Graças Vale Barbosa Guerra, João Marcos Bemfica Barbosa Ferreira

**Affiliations:** 1 Manaus AM Brazil Universidade do Estado do Amazonas – Programa de Pós-Graduação em Medicina Tropical, Escola de Ciências da Saúde, Manaus, AM – Brazil; 2 Manaus AM Brazil Fundação de Hematologia e Hemoterapia do Amazonas, Manaus, AM – Brazil; 3 Manaus AM Brazil Fundação de Medicina Tropical Doutor Heitor Vieira Dourado, Manaus, AM – Brazil; 4 Manaus AM Brazil Universidade Federal do Amazonas, Manaus, AM – Brazil; 5 São Paulo SP Brazil Beneficência Portuguesa de São Paulo, São Paulo, SP – Brazil

**Keywords:** Chagas Disease, Chagas Cardiomyopathy, Heart Failure

## Introduction

Chagas disease (CD) is caused by Trypanosoma cruzi, a protozoan with a complex life cycle found in dozens of species of triatomine insects (kissing bugs) and mammals.^1^ The World Health Organization (WHO) included CD among the neglected tropical diseases in 2006.^2^

Humans can become infected through contact with insect feces containing T. cruzi or through other mechanisms, including the ingestion of contaminated food. The clinical presentation of CD varies between its two phases: acute and chronic. In the acute phase, individuals are usually oligosymptomatic, presenting with nonspecific signs and symptoms. However, a minority may develop more severe manifestations such as hepatosplenomegaly, myocarditis, or meningoencephalitis. The chronic phase comprises three well-defined clinical forms: indeterminate, cardiac, and digestive.^1–6^

For many years, the Amazon region was considered free of endemic CD, but it is now the area with the highest number of acute cases, largely due to outbreaks of orally transmitted infection associated with the consumption of palm fruit juices, particularly açaí (*Euterpe oleraceae*). In the state of Amazonas, some cases of chronic Chagas cardiomyopathy (CCDC) have been diagnosed.^1,3^ This case report documents a 16-year longitudinal follow-up of the first autochthonous acute CD case monitored by the Chagas Disease Study Group at the Fundação de Medicina Tropical Dr. Heitor Vieira Dourado (FMT-HVD) in Amazonas. The patient's clinical course was followed from the initial diagnosis during the acute phase, through the various phenotypic manifestations of the cardiac form, culminating in clinical improvement.

## Case report

A 31-year-old man from Manaus, Amazonas (Brazil), was admitted to the FMT-HVD, a tertiary referral center, in 2007 with a history of fever, palpitations, shortness of breath, and chest pain after ingesting palm açaí juice.

Acute CD was diagnosed through direct parasitological examination using a thick blood smear. The electrocardiogram (ECG) showed frequent premature ventricular contractions, and the transthoracic echocardiogram (TTE) demonstrated mild left ventricular systolic dysfunction with an ejection fraction of 50%. The patient received benznidazole as etiological treatment for CD and was started on standard heart failure (HF) therapy, including an angiotensin-converting enzyme inhibitor, a beta-blocker, and a loop diuretic. However, after hospital discharge, he discontinued benznidazole after 30 days, failing to complete the full 60-day mandatory regimen. Several months later, his cardiac tests had normalized, allowing for the gradual withdrawal of HF medications.

He subsequently had irregular cardiology follow-up. In 2010, he returned to FMT-HVD reporting dyspnea on moderate exertion and palpitations. Serology (ELISA) for T. cruzi was negative, and two xenodiagnosis tests were also negative. The resting ECG showed sinus rhythm with isolated premature ventricular contractions and normal repolarization. Both TTE and cardiac magnetic resonance imaging were normal, while 24-hour Holter monitoring revealed three episodes of nonsustained ventricular tachycardia. An esophagogram demonstrated complete esophageal emptying. At that time, he was diagnosed with the arrhythmogenic form of chronic CCDC. Treatment with amiodarone led to clinical improvement.

He remained asymptomatic until 2015, when palpitations recurred. At that time, amiodarone and captopril were reintroduced. Repeat evaluations, including negative T. cruzi serology (ELISA) and cardiologic tests (ECG and exercise stress test), showed results within normal limits.

In 2018, 2019, and 2022, the patient returned for follow-up visits, during which he remained clinically stable with no significant changes in the cardiologic assessments, as summarized in Table 1.

**Table 1:** Cardiological exams performed on the patient during the follow-up period, 2007 to 2023

Year	Exam	Main Alterations
2007	Electrocardiogram	Sinus rhythm; premature ventricular contraction
	Echocardiogram	mild left ventricular systolic dysfunction; EF 50%
2010	Electrocardiogram	Sinus rhythm; premature ventricular contraction; normal repolarization
	Echocardiogram	Preserved ejection fraction, intracavitary dimensions normal
	Cardiac Magnetic Resonance imaging	LV cavity enlargement with no signs of fibrosis
	24-hour holter	Sinus rhythm, 28190 isolated, paired ventricular contraction, 3 episodes of non-sustained ventricular tachycardia
2018	Electrocardiogram	Sinus rhythm with frequent ventricular contractions
	Echocardiogram	mild enlargement intracavitary dimensions with LVDD 60 mm, LVEF (Simpson) 50%, with global mild systolic dysfunction
	24-hour holter	7668 isolated, interpolated, bigeminated, trigeminated, polymorphic ventricular contractions
	Cardiac Magnetic Resonanse	LV cavity enlargement with no signs of fibrosis
2019	Electrocardiogram	sinus rhythm, repolarization alteration in inferior wall
	Echocardiogram	Increased intracavitary dimensions; LVDD 60 mm; LVEF (Simpson) 53%
2022	Electrocardiogram	sinus rhythm, without alterations
	Echocardiogram	marked reduction in ejection fraction (LVEF 37%) and moderate left ventricular systolic and diastolic dysfunction
	24-hour holter	sinus rhythm with 193 isolated, interpolated, bigeminated, trigeminated, monomorphic ventricular extrasystoles
2023	Electrocardiogram	sinus rhythm, normal repolarization.
	Echocardiogram	LVEF (Simpson) 52%; LVEF 60 mm
	24-hour holter	Sinus rhythm, 444 isolated ventricular contraction

LVDD: left ventricular diastolic diameter; LVEF: left ventricular ejection fraction.

During this period, serologic tests for T. cruzi (ELISA: result 1.3, cut-off 0.27; IFA: 1/160) were reactive (Figure 1).

**Figure 1:** A) Electrocardiogram realized in 2020; B) 24h holter realized in 2020.

At the 2022 re-evaluation, the patient presented with clinical signs of HF, including exertional dyspnea and orthopnea. There was a significant reduction in left ventricular ejection fraction (LVEF 37%), indicating moderate systolic dysfunction. Serologic tests (ELISA and IFA) remained reactive. The 24-hour Holter monitor showed sinus rhythm with 193 isolated, interpolated, bigeminal, trigeminal, and monomorphic ventricular premature contractions. Guideline-directed medical therapy (GDMT) was initiated, including carvedilol 25 mg twice daily, enalapril 10 mg/day, spironolactone 25 mg/day, and dapagliflozin 10 mg/day. Despite one month of treatment, he continued to experience tachycardia and paroxysmal nocturnal dyspnea, leading to the addition of furosemide 40 mg/day and digoxin 0.25 mg/day.

In 2023, while continuing HF treatment, new tests were performed. The ECG showed sinus rhythm with a heart rate of 69 bpm. The TTE demonstrated a LVEF of 52% (Simpson) and a left ventricular diastolic diameter (LVDD) of 60 mm, meeting the criteria for HF with improved ejection fraction (Figures 2 and 3).

**Figure 2:** Post-treatment ECG's.

**Figure 3:** 24h Holter exam realized in 2023.

During the follow-up period, the patient was also diagnosed with cutaneous-mucosal leishmaniasis and pulmonary tuberculosis, both of which were treated according to clinical guidelines. The history of cardiologic examinations performed throughout this long-term follow-up is summarized in Table 1.

## Discussion

This case represents the first documented progression to CCDC in the state of Amazonas, Brazil. Over a 16-year follow-up, the patient initially developed an arrhythmogenic form of CCDC, later progressing to HF with reduced ejection fraction, and subsequently to HF with recovered ejection fraction. Previous studies have shown that antiparasitic treatment reduces both the proportion and the risk of symptomatic HF.^7–9^

However, the effectiveness of antiparasitic therapy in achieving complete parasite clearance varies, and higher parasite burden is associated with stronger serological responses. Despite more than 10 years of negative serologic tests in this case, cardiac manifestations persisted, suggesting ongoing disease activity. The long period of negative serology followed by later reactive results is noteworthy. Diagnostic variability in the Amazon region is well recognized, with a high frequency of false-negative results.^10^

Additionally, reinfection cannot be excluded, as the patient continued to live in an endemic area. Importantly, he did not complete trypanocidal treatment with benznidazole. In a previous study from the Brazilian Amazon involving 179 patients who completed benznidazole therapy, approximately 30% remained seropositive after seven years; however, only 5.2% showed electrocardiographic or echocardiographic abnormalities suggestive of CCDC.^11^

During the early phase of Chagas cardiomyopathy, premature ventricular contractions are the predominant arrhythmia. As the disease progresses and HF develops, about 99% of patients exhibit frequent, multiform, paired ventricular contractions and episodes of non-sustained ventricular tachycardia. Consistent with this pattern, the patient developed palpitations and ventricular arrhythmias and was classified as having CCDC with an arrhythmogenic phenotype.^3,8,9^

Fifteen years after diagnosis, the patient presented with HF characterized by a reduced ejection fraction (LVEF 37%), consistent with the natural progression of inadequately treated HF, which is associated with increased cardiovascular risk and reduced quality of life. His clinical course aligns with findings from a systematic review and meta-analysis showing a cumulative probability of cardiomyopathy of approximately 17% at 10 years and 31% at 20 years among individuals with CD.^12^

Adherence to guideline-directed medical therapy – including angiotensin-converting enzyme inhibitors, beta-blockers, mineralocorticoid receptor antagonists, and SGLT2 inhibitors – resulted in improved left ventricular function and a reduction in ventricular arrhythmias.^13^

The absence of fibrosis on cardiac MRI despite ventricular dysfunction is noteworthy. Previous studies from the Brazilian Amazon have shown that 58.3% of patients with acute CD exhibit myocardial fibrosis on MRI. However, these patients generally had a good prognosis and no symptoms of HF.^14^ This suggests that, in the patient described here, another pathophysiological mechanism – such as autonomic dysfunction – may have contributed to ventricular impairment, potentially explaining the rapid improvement with pharmacological therapy.

In a systematic review evaluating the risk of chronic cardiomyopathy among patients with acute or indeterminate forms of CD, case series from various regions of Latin America indicated an estimated annual cardiomyopathy incidence of 4.6% among individuals with acute infection, suggesting a relatively low rate of progression to chronic disease.^9^

This case highlights how neglected diseases such as CD disproportionately affect economically disadvantaged populations. Despite the availability of diagnostic and therapeutic resources for many conditions, a large portion of the Amazonian population continues to face diseases like CD, which thrive under conditions of poverty and perpetuate social inequities.^3^

In conclusion, there is an urgent need for improved understanding of CD in the Amazon, along with enhanced diagnosis and treatment of HF, to reduce disparities in healthcare access and address treatable causes of HF in the Amazonian population.

## Data Availability

The underlying content of the research text is contained within the manuscript.
